# Magnitude and factors associated with treatment non-adherence among patients with depressive disorders at St. Amanuel Mental Specialized Hospital, Addis Ababa, Ethiopia, 2019: A cross sectional study

**DOI:** 10.1371/journal.pone.0271713

**Published:** 2022-07-28

**Authors:** Yadeta Alemayehu, Henock Asfaw, Million Girma

**Affiliations:** 1 Department of Psychiatry, College of Health Science, Mettu University, Mettu, Ethiopia; 2 Department of Psychiatry, School of Nursing and Midwifery, College of Health and Medical Science, Haramaya University, Harar, Ethiopia; PLOS, UNITED KINGDOM

## Abstract

**Background:**

Inadequate adherence to treatment is among the main underlying causes of depression becoming a chronic problem. In developing countries due to limited access to health care, inaccurate diagnoses, and scarcity of medications, poor adherence may become an even larger obstacle in the treatment of depression. The current study aims to assess the magnitude and factors related to treatment non-adherence among patients with depressive disorders.

**Objective:**

To assess the magnitude and factors associated with treatment non adherence among patients with depressive disorders at St. Amanuel Mental Specialized Hospital, Addis Ababa, Ethiopia, 2019.

**Methods:**

A hospital-based cross-sectional study was conducted among 415 respondents using systematic random sampling technique. Medication adherence was assessed by using Medication Adherence Rating Scale. Data was entered to Epi-data version 3.1 and analyzed using SPSS version 20. Binary logistic analysis was done and P-values less than 0.05 were considered statistically significant.

**Results:**

The prevalence of treatment non-adherence among patients with depressive disorders was 26% (95%CI; 21.2, 32.5). Previous suicide attempt (AOR = 3.05, 95%CI; 1.82, 5.12), medication side effects (AOR = 2.46, 95%CI; 1.47, 4.11), moderate to high self-stigma (AOR = 2.60, 95%CI; 1.45, 4.66), and poor quality of life (AOR = 2.47, 95%CI; 1.42, 4.28) were significantly associated with treatment non-adherence among patients with depressive disorders.

**Conclusion and recommendation:**

Treatment non-adherence is a common problem among patients being treated for depressive disorders. Previous suicide attempts, medication side effects, moderate to high self-stigma, and poor quality of life were significantly associated with treatment non-adherence. Appropriate interventions should be developed to promote measures to facilitate adherence in this group of patients, and address the associated factors when applicable.

## Introduction

Treatment adherence is the extent to which a person’s behavior such as taking medications, following a diet, and/or executing lifestyle changes correspond with medical or health advice provided by health care providers [1]. Patients may choose not to fill their medications at the pharmacy and to forego starting treatment altogether, they may take more or less medication than advised, or they may take it at the wrong times. This may lead them to stop treatment early before the intended effect of the medication occurs [2, 3].

In low and middle income countries, in addition to poor access to health care, lack of diagnostic certainty, and scarcity of medications, poor adherence is a major challenge in the treatment of the common illnesses such as diabetes, depression, and HIV/AIDS [1].

Depression is a significant mental illness that affects more than 300 million people of all ages across the world. According to a World Health Organization report in 2017, depression was the third leading contributor to the worldwide burden of diseases and is predicted to become the second leading cause of the global burden of disease by the year 2030 [4, 5]. In Ethiopia, depressive disorders contribute to about 6.5% of the total burden of diseases, which is the highest share of burden in comparison to other mental disorders [6].

Patients with depressive disorders are better treated and recover earlier when they follow the provided medications according to the clinicians’ prescription[2]. Despite expectations, patients at times fail to follow prescribers’ instructions, either discontinuing antidepressant medications early, or taking a lower dose of the prescribed medication [7]. Given this reality, patient adherence is a critical aspect of effective clinical management [8].

Inadequate adherence to treatment is among the main underlying causes of depression becoming a chronic problem [9]. An untreated episode of depression lasts 6 to 13 months; most treated episodes of depression last about 3 months. The withdrawal of antidepressant medications before 3 months has elapsed often results in the return of the symptoms of the illness [10].

According to studies done in different parts of the world, the magnitude of treatment non adherence among patients with depressive disorders ranges from 10% to 67% [11–16]. A systematic review and meta-analysis study showed the magnitude of psychotropic medication non-adherence among patients with a diagnosis of major depressive disorder to be 50% [17].

According to a WHO report, the determinants of non-adherence are categorized in to different dimensions as: social and economic, health system-related, therapy-related, condition-related, and patient-related [1]. In another study determinants of treatment adherence were patient-specific, medication-specific, healthcare provision and system, social-cultural, and logistics factors [18].

Different studies show factors like duration of illness, severity of illness, previous history of admission, comorbid physical illness, lack of social support, presence of self-stigma, and substance use are correlates of treatment non-adherence among patients with depressive disorders [18–22]. In addition, those patients who have poor quality of life tend to be less adherent to their treatments [23].

There is a paucity of epidemiologically reliable data targeting magnitude and related factors of treatment non-adherence among patients with depressive disorders in Ethiopia, so this study will provide valuable data for clinicians, researchers and governmental bodies to address the problem.

## Method and materials

### Study design and period

A hospital based cross-sectional study was conducted from May 6 to June 13, 2019

### Study area

The study was conducted at St. Amanuel mental specialized hospital (St. AMSH). The hospital is the only specialized mental health hospital in the country and was established in 1930. It is located in the western part of Addis Ababa in Addis Ketema sub city. St. AMSH provides care for patients coming from the entire nation of Ethiopia.

### Source population

All patients with depressive disorders on follow up at St. Amanuel mental specialized hospital.

### Study population

Patients with depressive disorders who had a follow up visit at St. Amanuel mental specialized hospital during the study period.

### Sample size and sampling technique

The sample size was calculated by using single population proportion formula; since there was no published study in Ethiopia, the population proportion (P) was taken to be 50%. The sample size was calculated to be 384 participants. Considering, a 10% (39 participants) rate of non-response resulted in a total sample size of 423.

Systematic random sampling technique was used to select representative samples of patients with depressive disorders who had at least one follow up visit at the outpatient department of St. AMSH. The first participant was selected by lottery method then continued by every 2 intervals (k). The data was collected from every other patient with depressive disorders on follow up.

### Inclusion criteria

Adult (age≥18) patients with depressive disorders presenting for a follow up visit at St. Amanuel mental specialized hospital during the data collection period.

### Study measurements

**Medication adherence:** assessed using the Medication Adherence Rating Scale (MARS). A total score of less than 6 was considered non-adherent [24].

**Self-stigma:** Based on the 24-item Internalized Stigma of Mental Illness scale(ISMI), where a mean score of 1.00–2.00 indicates minimal to no self-stigma, a score of 2.01 to 2.50 indicates mild self-stigma, a score of 2.51 to 3.00 indicates moderate self-stigma, and a score of 3.01 to 4.00 indicates severe self-stigma [25].

**Social support:** Based on the Oslo social support scale. The total score ranges from 3–14 which will be interpreted as: [3–8] poor social support, [9–11] moderate social support, and [12–14] strong social support [26].

**Current substance use:** those who non-medically used at least one substance(alcohol, Khat, cigarette and others) within the last 3 months [27].

**Khat:** is a stimulant drug found in leaves of an East African shrub that contains cathinone and cathine.

**Suicide attempt:** The question on suicide was asked whether the participant had felt so desperate that they had attempted suicide and is recorded as yes or no [28].

**Quality of Life**: using EUROHIS-QOL 8-item index; which has a score ranging from 8 to 40; and the average score level was taken to classify participants into poor or good quality of life groups [29].

**Chronic depression:** if the duration of depressive disorder is greater than or equal to 2 year it’s considered to be chronic [30].

**Comorbid medical illness:** is a diagnosed medical or surgical problem. It was confirmed by reviewing patient’s chart and asking the patient.

**Severity of illness:** was assessed using CGI severity scale; scores 1–3 were considered as mild, 4 as moderate and 5–7 as severe depressive disorder [31].

**Level of Insight:** A total score of zero is regarded as "no insight", total score of 1–2 is "partial insight", and a total score of 3 is interpreted as "full insight" based on three adopted questions [32].

**Income:** Using the World Bank poverty line cutoff, those with an average monthly income of less than 1.9$/day were considered as low income [33].

### Data collection procedures

Data was collected by face-to-face interview, in addition to chart review. Socio-Demographic variables were collected using a semi-structured questionnaire.

Medication adherence was assessed using Medication Adherence Rating Scale. The total score of 6 or more indicate adherence and less than 6 indicate non adherence [24] The internal consistency (Cronbach’s alpha) of MARS in the current study is 0.86.

Severity of depression was assessed by using the improved Clinical Global Impression scale which has an objective CGI question that was filled by observing clinician and subjective CGI questions that was answered by participants [31].

For the assessment of insight the following dichotomous questions were asked and a score of 1 is assigned for yes and a score of zero for no for any item answered: Do you accept that you have an illness? Do you think that you require treatment? And, do you think you require your medications to stay well? The total score ranges between 0 and 3. A total score of zero is regarded as "no insight", total score of 1–2 is "partial insight", and a total score of 3 is interpreted as "full insight" [32].

Quality of Life was assessed using EUROHIS-QOL 8-item index. It’s prepared by WHO and validated in patients with depressive disorder. It has eight questions that were answered by participants and it was taken as poor quality of life if average score level was<24 and good quality of life if average score was ≥24 [29].

### Data quality control

All questionnaires was translated into Amharic and back translated into English to check its consistency. The questionnaires were pretested at St. Paul’s Millennium Medical College Hospital on 5% (n = 22) of sample size that was not included in the main study. Data collectors were trained on the questionnaires and data was collected by psychiatric nurses. Data collectors were supervised daily and the filled questionnaires were checked daily by the supervisor and principal investigator.

### Data processing and analysis

Coded and checked data were entered into the computer using EPI Data version 3.1 and imported to statistical package for social science (SPSS) window software version 20. Descriptive statistics such as frequency, percentage, mean and median were computed and presented using tables and charts in the current manuscript. Bi-variable binary logistic analysis was performed to determine each of explanatory variables and variables with p- value less than 0.2 during bi-variable analysis were entered to multivariable analysis. Multivariable binary logistic regression analysis was conducted to determine the presence of a statistically significant association between explanatory variables and outcome variables. P values less than 0.05 were considered statistically significant and strength of the association was presented by adjusted odds ratio with 95% C.I.

### Ethical consideration

Ethical clearance was obtained from ethical review committee of St. Amanuel mental specialized hospital. Formal letter of permission was obtained from St. Amanuel mental specialized hospital. Data collectors clearly explained the aims of the study to study participants. Study participants had the opportunity to refuse or discontinue participation at any time during the course of the study, and they were further given the chance to ask any thing about the study. Data was collected after obtaining written consent from participants. Confidentiality was assured throughout the study period.

## Results

### Socio-economic and demographic characteristics of respondents

Of the 423 total sample size, responses were received from 415, yielding a response rate of 98.11%. Eight patients failed to complete the expected questionnaires; six of them were due to lack of sufficient time for an interview, and two of them became agitated after initiating the interview. The respondents’s mean age was 36 years (standard deviation (SD): 10.11). Females made up 220 (53%) of the participants. More than half of the participants (227(54.7%)) were Orthodox, almost half of the respondents (187(45.1%)) were married, and 123 (29.6%) had completed college. About 250 (60.24%) were employed, and 314 (75.7%) were city dwellers. **(Table 1).**

**Table 1:** Socio-economic and demographic characteristics of study participants.

Variables	Frequency(n = 415)	Percentage (%)
Sex		
Female	220	53.02
Male	195	46.98
Age		
18–24	56	13.49
25–34	130	31.33
35–44	121	29.16
45–54	65	15.66
≥55	43	10.36
Religion		
Christian	308	74.22
Muslim	107	25.78
Marital status		
Married	187	45.06
Single/divorced/widowed	228	54.94
Occupation		
Employed	250	60.24
Unemployed	165	39.76
Education status		
No formal education	70	16.86
Primary education	111	26.75
Secondary education	111	26.75
Tertiary and above	123	29.64
Residency		
Urban	314	75.66
Rural	101	24.34
Average income		
<1.9$/day	278	66.98
≥1.9$/day	137	33.02

### Respondents’ clinical characteristics

More than half (54.6%) of participants have had multiple episodes of the illness. A slight majority, 292(70.4%), had chronic depressive disorders, 215(51.81%) had full insight, and 34.46% of respondents have attempted suicide at some point in their lives. 55.2% of respondents had moderate severity of depression. Using EUROHIS-QOL 8-item the mean quality of life score in this study was 23.53 (SD 3.12). **(Table 2).**

**Table 2:** Clinical characteristics of the study participants.

Variables	Frequency	Percentage (%)
Episode		
First Episode	181	43.61
Multiple episode(≥2)	234	56.39
Chronicity of depression		
Chronic	292	70.36
Non-chronic(acute)	123	29.64
Levels of Insight		
No Insight	55	13.25
Partial Insight	145	34.94
Full Insight	215	51.81
Comorbid medical condition		
Yes	105	25.30
No	310	74.70
Suicidal attempt		
Yes	143	34.46
No	272	65.54
Severity of depression		
Mild	146	35.18
Moderate	229	55.18
Severe	40	9.64
Level of stigma		
No/Minimal	86	20.73
Mild	190	45.78
Moderate	119	28.67
Severe	20	4.82
Level of Social support		
Poor	166	40.00
Moderate	180	43.37
Strong	69	16.63
Quality of life		**23.53 ± 3.12SD**

**→**SD = standard deviation

### Current substance use history of respondents

**Fig 1:**
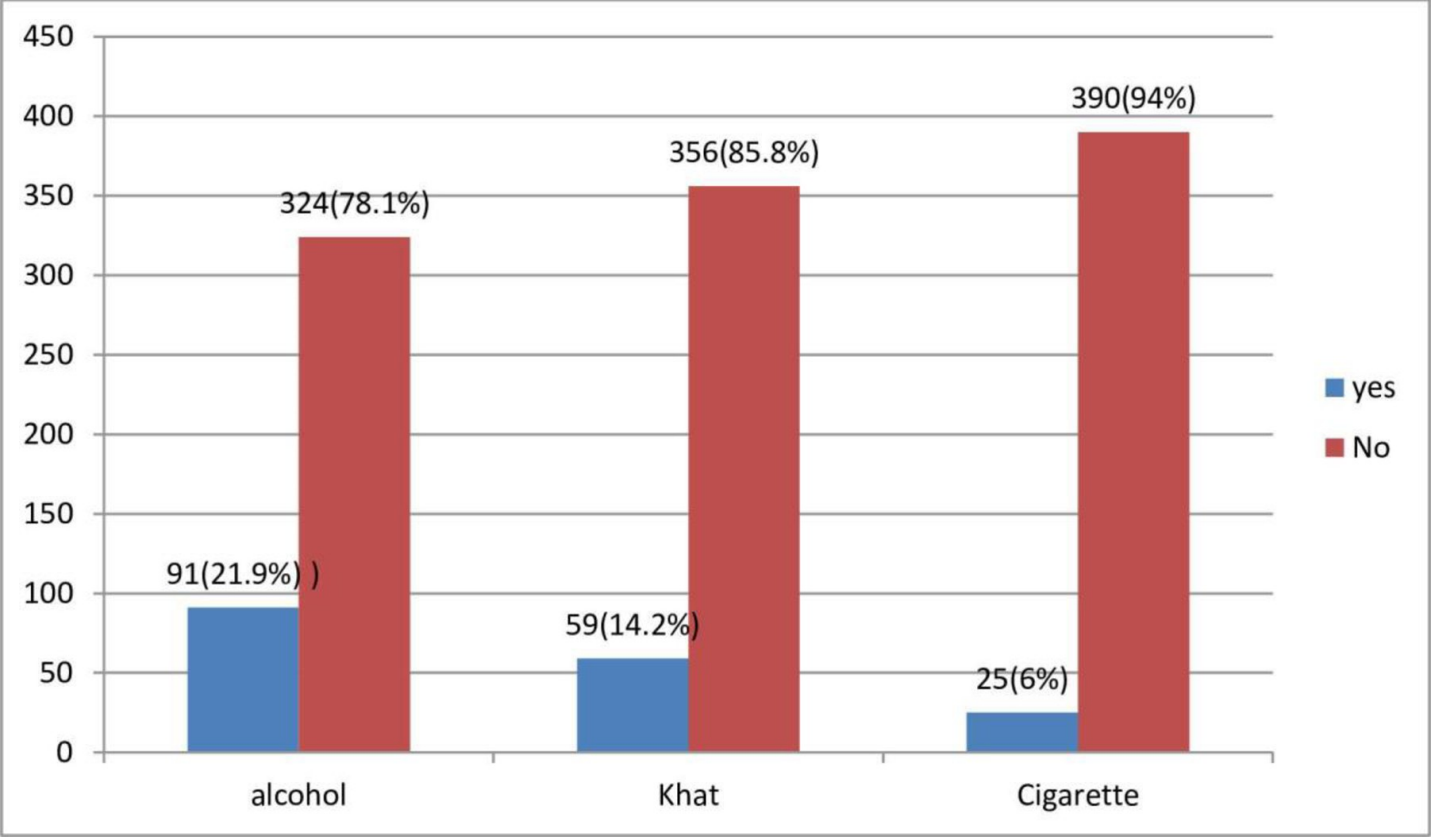
Current substance use characteristics of participants. *→NB*:*-* No other addictive substances were reported.

From the total of 415 respondents 91(21.9%) used alcohol, and 59(14.2%) used Khat within three months prior to data collection time **(Fig 1).**

### Magnitude of treatment non-adherence

**Fig 2:**
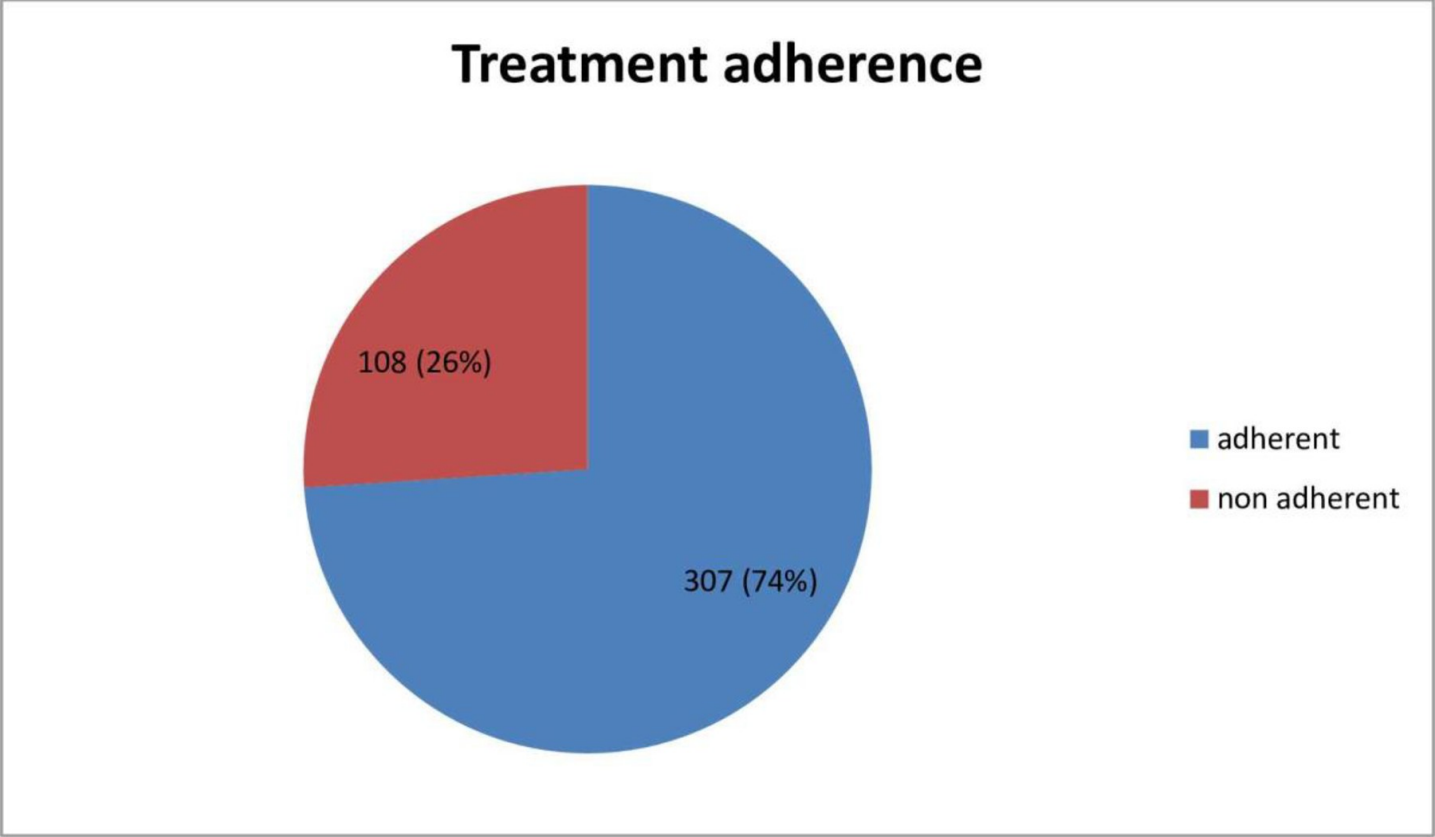
Magnitude of treatment non adherence among study participants.

In this study about one fourth of respondents 108(26%) were non adherent to their treatment (**Fig 2**).

### Factors associated with treatment non adherence

Being female, single, having low-income, having multiple episodes of depression, experiencing medication side effects, history of suicidal attempt, level of self-stigma, limited social support, and poor quality of life were variables with p-values less than 0.2 in bivariable binary logistic regression.

Medication side effects, history of suicidal attempt, poor social support, poor quality of life, and moderate to high levels of self-stigma were found to have a statistically significant association with medication non-adherence at a p-value less than 0.05 in multivariable binary logistic regression variables.

The odds of being non adherent was 3.05 times greater among respondents with a lifetime history of suicidal attempt than among those without a history of suicidal attempt (AOR = 3.05, 95%CI; 1.82, 5.12).

When compared to those who did not have medication side effects, the odds of being non adherent was 2.46 times higher among those who had medication side effects(AOR = 2.46,95%CI;1.47, 4.11).

The odds of being non adherent was 2.6 times higher among those with moderate to high self-stigma compared to those with minimum to low self-stigma (AOR = 2.60,95%CI;1.45, 4.66).

When comparing respondents with poor quality of life to those with good quality of life, the odds of being non adherent was 2.47 times higher (AOR = 2.47,95%CI; 1.42, 4.28) **(Table 3)**.

**Table 3:** Bivariable and multivariable binary logistic regression analysis showing association between treatment non adherence and related factors among study participants.

Explanatory variables	Treatment non adherence		COR (95%CI)	AOR (95%CI)
	yes	No		
Sex				
Female	70	150	1.93(1.23, 3.04)	1.70(0.96, 3.01)
Male	38	157	1	1
Marital status				
Married	38	149	1	1
Single	50	86	2.28(1.39, 3.75)	1.79(0.97, 3.29)
Divorced/widowed	20	72	1.09(0.56, 2.01)	1.57(0.79, 3.10)
Income				
<1.9$/day	81	197	1.67(1.02, 2.33)	1.26(0.68, 2.36)
≥1.9$/day	27	110	1	1
Episode				
First	47	186	1	1
Multiple	61	121	1.99(1.28, 3.11)	1.38(0.79, 2.39)
Suicidal attempt				
Yes	62	81	3.76(2.38, 5.95)	**3.05(1.82, 5.12)** *
No	46	226	1	1
Severity of depression	30	116	1	1
Yes	63	166	0.68(0.42,1.12)	1.14(0.64,2.05)
No	15	25	0.43(0.20,0.92)	1.15(0.44,2.98)
Treatment side effect				
Yes	64	101	2.97(1.89, 4.66)	**2.46(1.47,4.11)** *
No	44	206	1	1
Level of stigma				
No/mild	43	233	1	1
Moderate to high	65	74	4.76(2.99,7.58)	**2.60(1.45, 4.66)** *
Social support				
Poor	55	111	2.13(1.08, 4.23)	0.93(0.49,1.76)
Moderate/Strong	53	196	1	1
Quality of life				
Poor	75	131	3.05(1.91, 4.87)	**2.47(1.42, 4.28)** *
Good	33	176	1	1

→ * indicates statistically significant association, Hosmer and Lemeshow Test = .748

## Discussion

In the current study the frequency of treatment non adherence among patients with depressive disorder was 26% (95% CI; 21.9%-30.6%). This finding is consistent with previous studies done in Spain (30.1% [22]), in United Kingdom (30% [16]) and in USA (28% [34]).

However, it was lower than studies done in Nigeria (55.7% [32]), Saudi Arabia (52.9% [14]), India (66.9% [11]), Japan (63.1%([12]), and Thailand (59%[13]). The possible reason for this discrepancy might be the differences in populations and scales. For instance, the Nigerian study used Morisky medication adherence rating scale and the was among patients with severe mental illness not specific to depression; similarly the study in Saudi Arabia used Morisky medication adherence rating scale and the and had a 6 months minimum duration of treatment in the Saudi Arabian study; this longer duration might affect adherence rate as patients might lose confidence in a treatment if their illness has not improved during this time, causing them to quit the treatment. The studies done in India and in Japan used Morisky Medication Adherence Scale and medication possession rate respectively, unlike the one used in the current study (MARS). The study in Thailand also used medication possession ratio which varies from the current study.

However, the frequency of non-adherence was lower in the study done in United Kingdom 10% [15]. The possible reason might be the difference in age groups as the UK study only included participants above 50 years but the current study included all adults. The other possible reason is the difference in socio-economic status; accessibility and affordability of medication and minimal side effects can explain the improvements in adherence [35].

Those who had attempted suicide in their life were 3.05 times more likely to be non-adherent than those who had not attempted suicide. This is supported by studies done in India [36] and in Czech Republic [21] This association might have occurred because depressive disorders are the main underlying cause of suicidal behavior among mental disorders. If depression is not appropriately treated due to non-adherence, suicidal behavior as a symptom of depression will remain untreated [36, 37].

Patients who had medication side effects were 2.46 times more likely to be non-adherent than those who don’t have medication side effects. This association is supported by a study done in Malaysia [18] and other systematic reviews in Spanish and English languages [22]. The association might be related to intolerance of the medication adverse effects, thinking that the side effects of the medications are harmful; in addition a prescriber’s inadequate knowledge and skill in managing the illness and related adverse effect can have an impact and cause patients to prematurely stop medications [38].

Patients with moderate to high self-stigma were 2.6 times more likely than those with minimal to mild self-stigma to be non-adherent to the treatment. This association is consistent with studies done in Malaysia [18], Czech Republic [21] and USA [19] The possible reason for the association might be those who were non adherent to the treatment have poor ability to control or recover from the illness, worsening their level of self-stigma [39].

Respondents who reported a poor quality of life were 2.47 times more likely than those who reported a good quality of life to be non-adherent to the treatment. This finding is consistent with the survey done by EU National Health and Wellness Survey (NHWS) [23]. The other possible reason for the association might be that treatment non-adherence can cause a worsening of the illness; and can increase social isolation which is major reflection of poor quality of life. The other reason for the association might be that if a patient with chronic medical disorders including depression fails to adhere to treatment due to their physical, social and psychological health issues, which are indicators of poor quality of life [40, 41].

Although factors like chronicity of depression, severity of depression, number of episodes, comorbid medical illness, lack of social support, level of insight, educational status and current substance use were associated with treatment non adherence in different studies, but they failed to have statistically significant association in the current study. The reason for this variation might be the difference in socio-cultural variations and tools which were employed in the current study.

The limitation of this study is the use of cross-sectional study design, so the current study is unable to show a temporal causal relationship between treatment non-adherence and major associated factors.

## Conclusion and recommendation

Treatment adherence is a common problem among patients on treatment for depression. Having a history of suicidal attempt, medication side effect, moderate to high self-stigma and poor quality of life were significantly associated with treatment non adherence. Suitable interventions should be developed to address these factors, and promote measures to facilitate adherence in this group of patients.

## Abbreviations

AOR: Adjusted Odd Ratio
CGI: Clinical Global Impression scale
CI: Confidence Interval
COR: Crude Odd Ratio
EUROHIS-QOL: European Health Interview Survey Quality of Life
ISMI: Internalized Stigma of Mental Illness
MARS: Medication Adherence Rating Scale
SPSS: Statistical Package for Social Science
St. AMSH: Saint Amanuel Mental Specialized Hospital
WHO: World Health Organization
